# Dietary Patterns, Their Nutrients, and Associations with Socio-Demographic and Lifestyle Factors in Older New Zealand Adults

**DOI:** 10.3390/nu12113425

**Published:** 2020-11-08

**Authors:** Karen Mumme, Cathryn Conlon, Pamela von Hurst, Beatrix Jones, Welma Stonehouse, Anne-Louise M. Heath, Jane Coad, Crystal Haskell-Ramsay, Jamie de Seymour, Kathryn Beck

**Affiliations:** 1 College of Health, Massey University, Auckland 0632, New Zealand; k.mumme@massey.ac.nz (K.M.); c.conlon@massey.ac.nz (C.C.); p.r.vonhurst@massey.ac.nz (P.v.H.); j.deseymour@massey.ac.nz (J.d.S.); 2 Department of Statistics, University of Auckland, Auckland 1010, New Zealand; beatrix.jones@auckland.ac.nz; 3 Health and Biosecurity Business Unit, Commonwealth Scientific Industrial Research Organisation (CSIRO), Adelaide, South Australia 5000, Australia; welma.stonehouse@csiro.au; 4 Department of Human Nutrition, University of Otago, Dunedin 9016, New Zealand; anne-louise.heath@otago.ac.nz; 5 College of Sciences, Massey University, Palmerston North 4474, New Zealand; j.coad@massey.ac.nz; 6 Department of Psychology, Northumbria University, Newcastle NE1 8ST, UK; crystal.haskell-ramsay@northumbria.ac.uk

**Keywords:** dietary patterns, nutrient intakes, socio-demographic factors, principal component analysis, older adults, diet quality, education, sex differences, age, living alone, deprivation index, physical activity, alcohol, smoking

## Abstract

Dietary patterns analyse combinations of foods eaten. This cross-sectional study identified dietary patterns and their nutrients. Associations between dietary patterns and socio-demographic and lifestyle factors were examined in older New Zealand adults. Dietary data (109-item food frequency questionnaire) from the Researching Eating, Activity and Cognitive Health (REACH) study (*n* = 367, 36% male, mean age = 70 years) were collapsed into 57 food groups. Using principal component analysis, three dietary patterns explained 18% of the variation in diet. Dietary pattern associations with sex, age, employment, living situation, education, deprivation score, physical activity, alcohol, and smoking, along with energy-adjusted nutrient intakes, were investigated using regression analysis. Higher ‘Mediterranean’ dietary pattern scores were associated with being female, higher physical activity, and higher education (*p* < 0.001, R^2^ = 0.07). Higher ‘Western’ pattern scores were associated with being male, higher alcohol intake, living with others, and secondary education (*p* < 0.001, R^2^ = 0.16). Higher ‘prudent’ pattern scores were associated with higher physical activity and lower alcohol intake (*p* < 0.001, R^2^ = 0.15). There were positive associations between beta-carotene equivalents, vitamin E, and folate and ‘Mediterranean’ dietary pattern scores (*p* < 0.0001, R^2^ ≥ 0.26); energy intake and ‘Western’ scores (*p* < 0.0001, R^2^ = 0.43); and fibre and carbohydrate and ‘prudent’ scores (*p* < 0.0001, R^2^ ≥ 0.25). Socio-demographic and lifestyle factors were associated with dietary patterns. Understanding relationships between these characteristics and dietary patterns can assist in health promotion.

## 1. Introduction

Non-communicable diseases are a large contributor to the global burden of disease in the ageing population, so it is important to understand the role of associated and modifiable risk factors, such as nutrition, that may minimise this burden [1,2]. Dietary pattern analysis explores the complete diet, complementing the traditional single food or nutrient approach [3], and is commonly used to examine diet–disease associations e.g., bone mineral density [4], cognitive health [5], and sarcopenia [6] in older adults.

There are two main approaches to dietary pattern analyses. A hypothesis driven approach (a priori) uses a pre-defined scoring system, often based on dietary guidelines, to determine adherence to a diet e.g., the Healthy Eating Index [7]. The second approach is data driven (a posteriori), reducing the dimensionality of many food groups to a few patterns while keeping as much variability within the diet as possible. Using dietary data from the study population, the a posteriori approach characterises the diet and eating habits specific to the study population rather than relying on current knowledge, as with the a priori approach [8].

Several studies in older adults have explored dietary patterns and associated socio-demographic and lifestyle factors. Higher education, income, and physical activity have consistently been associated with ‘healthy’, ‘vegetable based’, and ‘prudent’ dietary patterns [9,10,11,12,13,14,15,16,17,18], whereas smoking is associated with ‘Western’, ‘junk’, and ‘traditional/white bread’ dietary patterns [10,11,14,15,16,17]. In a New Zealand population of adults (15+ years), associations between socio-demographic factors and ‘healthy’ and ‘traditional’ dietary patterns were found [19]. Age was positively associated with both a ‘healthy’ and ‘traditional’ dietary pattern, therefore, more research to understand the specific dietary patterns of the older New Zealand population would be of interest. Ageing is associated with a number of physiological, psychological, and other changes, including loss of functionality, changes in living situation, e.g., loss of spouse, and possible dietary changes to support a health condition, such as lowering blood pressure [2]. Older populations also have distinct challenges and dietary needs e.g., higher calcium requirements [20], and therefore, may have their own unique dietary patterns compared with the general population. There is limited research investigating the dietary patterns of older adults living in New Zealand. Targeting nutrition interventions based on demographics may improve dietary intervention outcomes, especially when the demographics are specific to a sub-group of a population [19].

The aim of this study was to identify and describe the dietary patterns in an older, community-dwelling New Zealand population, including the nutrient differences across the dietary patterns, and to examine associations between dietary patterns and socio-demographic and lifestyle factors.

## 2. Materials and Methods

### 2.1. Study Design and Participants

The REACH (Researching Eating, Activity and Cognitive Health) study is a cross-sectional study that aims to explore associations of a posteriori dietary patterns with cognitive function and metabolic syndrome in older adults. The protocol and methodology of the REACH study have been published [21,22] and are outlined here. The study population was a convenience sample, which included 65 to 74 year old men and women living independently (i.e., in the community) in Auckland, New Zealand. Exclusion criteria included a diagnosis of any condition which may impair cognitive function or any event in the previous two years which may impact dietary intake. Informed written consent was obtained from all REACH participants. Massey University Human Ethics Committee granted ethical approval: Southern A, Application 17/69. All participants visited the Massey University Human Nutrition Unit in Auckland on one occasion.

### 2.2. Socio-Demographic and Lifestyle Data

Socio-demographic and lifestyle data were collected by written questionnaire during the visit to the Human Nutrition Unit. A written questionnaire captured data about age, sex, ethnicity, highest education level, work situation (employed or volunteering, not working), living situation (alone, with others), deprivation score [23], food insecurity [24], physical activity [25], smoking status, and alcohol beverage intake.

The New Zealand Indices of Multiple Deprivation and the participant’s residential address determined the area deprivation score based on seven domains: employment, income, crime, housing, health, education, and geographical access [23]. Eight indicator statements specific to a New Zealand population determined the level of food insecurity [24]. The International Physical Activity Questionnaire (short form) [25] measured physical activity levels. A physical activity score was calculated using metabolic equivalent of a task (MET-minutes), where one minute of activity is 3.3, 4.0, or 8.0 MET-minutes depending on exercise level: walking, moderate activity, and vigorous activity, respectively. One MET is the rate of energy expended while at rest [25]. Alcohol beverage intake (g/day) was calculated from the 109-item Food Frequency Questionnaire (FFQ) described below.

### 2.3. Dietary Assessment

Dietary data were collected between April 2018–February 2019 using an online 109-item FFQ representing the previous month’s diet. The FFQ has been shown to have acceptable validity and reproducibility for determining dietary patterns [22] and nutrient intakes [26]. Daily intake (g/day) of each food item was calculated using frequency and serving sizes from the FFQ. The 10 frequency choices were “I never eat this food”, “Not this month but I have sometimes”, “1–3 times per month”, “Once per week”, “2–3 times per week”, “4–6 times per week”, “once per day”, “2–3 times per day”, “4–5 times per day”, and “6 plus times per day”. Portion sizes were guided by FOODfiles, the New Zealand Food composition database [27]. Energy and nutrient values for each food item for each participant were calculated using the FOODfiles database [27] based on a representative food within that food item. For example, edam cheese represented the ‘cheese’ food item. Where necessary, a composite of foods was selected to represent the food item, e.g., ‘bran-based cereals’ was based on muesli, porridge, and sultana cereal. Average daily energy intake was considered implausible if < 2100 kJ (500 kcal) or > 14,700 kJ (3500 kcal) for women and < 3360 kJ (800 kcal) or > 16,800 kJ (4000 kcal) for men [22,28]. All nutrient values were adjusted for energy intake using the residual method [29].

The daily intake of the 109 food items was collapsed into 57 food groups for dietary pattern analysis. Four members of the research team decided the food groups based on similarity of foods, their nutrient profile, and culinary use, e.g., nuts and seeds are eaten in similar circumstances [30] (Table 1).

### 2.4. Construction of the Dietary Patterns

Based on correlations between food groups, principal component analysis reduces the dimensionality of food groups while retaining most of the variation within the diet. The data set was checked for suitability for principal component analysis using the Bartlett’s test of sphericity, measuring the presence of relationships within the data, and the Kaiser–Meyer–Olkin, which measures the sampling adequacy.

Using R version 3.6.1 [31], the principal() function in the psych package [32], and orthogonal varimax rotation (for ease of interpretation), dietary patterns from the data matrix of the 57 food groups (g/day) were derived from the FFQ. The factors (dietary patterns) retained were based on the scree plot, eigenvalues > 1.0, and interpretability. Factor loadings for each food group represented the correlation between the factor (dietary pattern) and the food group. A factor loading ≥0.30 or ≤ −0.30 was considered significant for this sample size [33]. Dietary pattern names were based on food groups with higher loadings and the diet that the food groups typified. Standardised dietary pattern scores were calculated for each participant for each dietary pattern using the regression method.

### 2.5. Statistical Analysis

Statistical analysis was performed using R version 3.6.1 [31] and R packages: tidyverse [34], car [35], and s20x [36]. Equality of variance and normality of residuals for regression models were assessed visually by graphing residuals and fitted values. No data were transformed prior to statistical analysis.

Participant characteristics were described by mean ± standard deviation for continuous characteristics, with a roughly symmetric distribution; median (25, 75 percentile) for other continuous data; or number and percentage for categorical data. The Welch two-sample t-test or Pearson chi-squared test examined differences between the sexes for characteristic variables. With relatively large sample sizes in each group (women: *n* = 235, men: *n* = 132), the group means had approximately normal distributions as required by the t-test. Only categorical variables with adequate samples in each category were considered for the Chi-squared test. As the population was homogenous in terms of ethnicity and food security (Table 2), these two variables were not included in association analyses.

Linear regression was used to determine associations between energy adjusted nutrients (residuals method [29]) and dietary patterns. The adjusted R^2^ was used to characterize the effect size of the associations. As multiple statistical tests were performed (*n* = 96), Bonferroni adjustments were made where the *p*-values were multiplied by the number of tests. Adjusted *p*-values < 0.05 were considered significant.

Multiple linear regression analysis was used to investigate associations between each dietary pattern score (dependent variable) and socio-demographic and lifestyle factors (independent variables). These included sex (male, female), age (years), physical activity score (tertiles), education (secondary, post-secondary, university), employment status (yes, no), living situation (alone, with others), index of multiple deprivation (score), alcohol consumption (g/day alcohol beverage intake), and smoking status (current or past, no). Variables in the full regression model were checked for collinearity using the variance inflation factor [35]. Scores ranged from 1.01 to 1.23, and no variables were considered collinear. Sex interactions were tested for each categorical independent variable. The full regression model included all independent variables plus significant interaction terms. Using a backwards stepwise process, the term with the largest *p*-value was removed until all independent variables were significant. As several statistical tests were performed, a *p*-value < 0.01 was considered statistically significant.

## 3. Results

### 3.1. Participants

A total of 371 participants took part in the REACH study. Four people were excluded due to not providing FFQ data. All participants had energy intakes within plausible parameters [22,28]. Most participants were New Zealand European and other (94%), and almost all were considered to be food secure (96%). Table 2 presents participant characteristics. Males were significantly older (*p* < 0.01), and more likely to have a university education (*p* < 0.001) and to live with others (*p* < 0.0001). They also consumed more alcohol beverages (*p* < 0.0001) and had a higher energy intake (*p* < 0.01) than females.

### 3.2. Dietary Patterns

Principal component analysis identified three dietary patterns from the FFQ data, which explained 18% of the variation in dietary intake. The Kaiser–Meyer–Olkin measure of sampling adequacy was 0.66, and Bartlett’s test of sphericity was significant (*p* < 0.0001), indicating the dietary data set was suitable for principal component analysis. Table 3 displays the dietary pattern loadings, range of dietary pattern scores, eigenvalues, and the variance explained by each dietary pattern.

**Figure 1:**
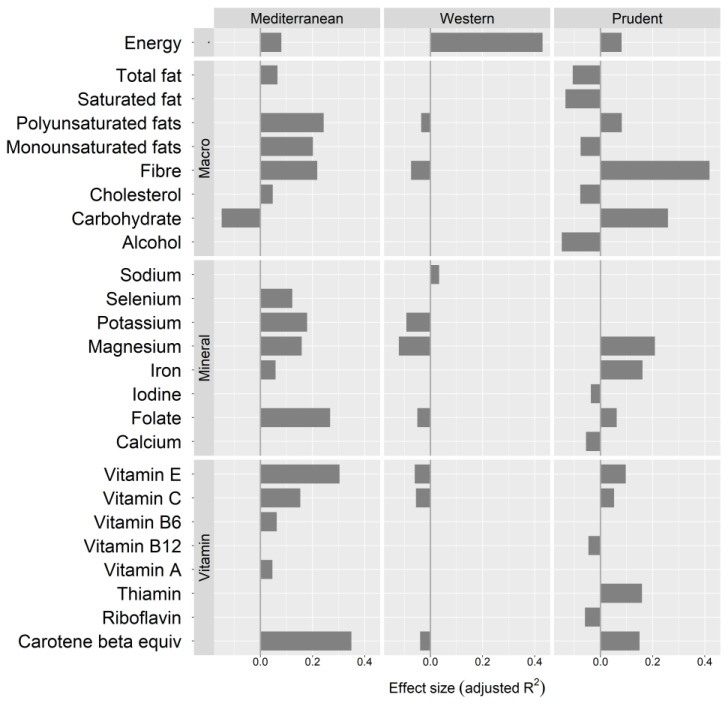
The plot shows the effect size of correlations between nutrients and each dietary pattern i.e., linear change as dietary pattern scores increase. Nutrients are adjusted for energy intake using the residual method [29]. Bars to the right of zero show a positive nutrient intake correlation to dietary pattern scores, and bars to the left show a negative nutrient intake correlation to dietary pattern scores. The size of the bar shows the magnitude of the effect (adjusted R^2^). All nutrients shown are significant after Bonferroni adjustment (adjusted *p*-value < 0.05). Protein, sugar, zinc, phosphorus, retinol, niacin, and niacin equivalent were analysed but showed no associations.

Dietary pattern 1 included ‘Mediterranean’ food groups. Positive loadings (≥0.30) were ‘salad vegetables’, ‘leafy cruciferous vegetables’, ‘other vegetables’, ‘avocados, olives’, ‘alliums’, ‘nuts, seeds’, ‘white fish, shellfish’, ‘oily fish’, ‘berries’, ‘water’, ‘salad dressings’, ‘cruciferous vegetables’, ‘eggs’, ‘cheese’, ‘tomatoes,’ and ‘all other fruit’ (Table 3). The ‘Mediterranean’ dietary pattern was positively associated with energy, polyunsaturated and monounsaturated fats, fibre, total fat, cholesterol, folate, potassium, magnesium, selenium, iron, beta-carotene equivalents, vitamin A, vitamin E, vitamin C, and vitamin B6. Negative associations were observed with carbohydrate (Figure 1).

Dietary pattern 2 included ‘Western’ food groups. Positive loadings (≥0.30) were ‘processed meats’, ‘sauces, condiments’, ‘cakes, biscuits and puddings’, ‘meat pies, chips’, ‘processed fish’, ‘confectionery’, ‘vegetable oils’, ‘salad dressings’, ‘beer’, ‘chocolate’, ‘cheese’, and ‘sweetened cereal’ (Table 3). The ‘Western’ dietary pattern was positively associated with energy and sodium intake, and negatively associated with fibre, polyunsaturated fats, magnesium, potassium, folate, vitamin E, vitamin C, and beta-carotene equivalents (Figure 1).

Dietary pattern 3 included ‘prudent’ food groups. Positive loadings (≥ 0.30) were ‘dried legumes’, ‘soy-based foods’, ‘fresh, frozen legumes’, ‘wholegrains’, ‘carrots’, and ‘spices’ (Table 3). The ‘prudent’ dietary pattern was positively associated with energy, fibre, carbohydrate, polyunsaturated fats, magnesium, iron, folate, thiamine, beta-carotene equivalents, vitamin E, and vitamin C and negatively associate with alcohol, saturated fat, total fat, cholesterol, monounsaturated fat, calcium, iodine, riboflavin, and vitamin B12 (Figure 1).

Protein, sugar, zinc, phosphorus, retinol, and niacin equivalents were not associated with any dietary pattern. No interactions between sex and dietary pattern to nutrients were present.

A validation study using a subset of the REACH study (*n* = 294) found dietary patterns obtained from the validated REACH FFQ to be reproducible and valid [22]. Additionally, the dietary patterns obtained from the validation study were comparable to those found in this manuscript. Tucker’s congruence coefficient (phi) between the loadings of the FFQ derived dietary patterns (REACH FFQ validation subset vs. REACH full cohort) were 0.96, 0.91, and 0.88 for ‘Mediterranean’, ‘Western’, and ‘prudent’ patterns, respectively.

### 3.3. Dietary Patterns and Socio-Demographic and Lifestyle Factors

The ‘Mediterranean’ pattern was positively associated with being female and having a higher physical activity tertile and a higher education (i.e., post-secondary or university). The ‘Western’ pattern was positively associated with being male, having a higher alcohol intake, and living with others. For male, secondary education predicted higher adherence to the ‘Western’ pattern compared with post-secondary or university education. This was not true for females (interaction, *p* < 0.01).

The ‘prudent’ pattern was positively associated with a higher level of physical activity and lower alcohol intake (Table 4).

## 4. Discussion

In our study of community dwelling older adults living in Auckland, New Zealand, three dietary patterns were identified: ‘Mediterranean’, ‘Western’, and ‘prudent’. Positive associations were found between physical activity and both patterns containing healthy food groups i.e., ‘Mediterranean’ and ‘prudent.’ Females were more likely to adhere to the ‘Mediterranean’ pattern and males to the ‘Western’ pattern. Education (positive association with the ‘Mediterranean’ pattern, negative association with the ‘Western’ pattern), alcohol consumption (positive association with ‘Western’, negative association with ‘prudent’), and living alone (negative association with ‘Western’) were all associated with at least one dietary pattern.

There are different approaches to analysing sex in dietary pattern analysis, one being to derive separate patterns for men and women. The other, as followed in this study, is to derive combined sex dietary patterns with sex as a variable in the statistical analysis. The low dimensional summary of sex differences produced in this study makes the second approach more favourable. However, within this study, women were more likely to adhere to the ‘Mediterranean’ and men to the ‘Western’ dietary pattern. Men and women are known to eat differently, and women have been shown to eat more fruit and vegetables than men in a general population [37,38], and in a 51 to 70 year old New Zealand population (but not 71+ years) [39]. This may be due to women having greater nutrition knowledge [37,40]. However, studies in older adults do not always show a defined trend between sex and dietary patterns, as older women may follow ‘vegetable-based’ [9], ‘fruit and milk’ [14], ‘sweet and fat dominated’ [9] or ‘Western’ [11] patterns, and men may follow ‘fat and meat’ [41] or ‘prudent’ [42] patterns. The Three-City and NuAge studies did not find any sex differences in ‘healthy’, ‘traditional’, or ‘Western’ patterns [11].

Sex interactions between dietary patterns and socio-demographic or lifestyle factors are either not reported or not commonly examined. In this study, we found a significant sex interaction with education (*p* < 0.01) when predicting the ‘Western’ pattern score. Having only a secondary education predicted a higher ‘Western’ score in men than women. In contrast, higher education predicted a higher ‘Mediterranean’ score in both men and women. Higher education, an important determinant to eating a nutritious diet [11,43,44,45], may bring better nutrition knowledge and an ability to earn a higher income allowing an opportunity to purchase healthier foods [46]. Dietary pattern and education associations (excluding the sex interaction) found in the current study are consistent with other studies in older adults. ‘Mixed’, ‘fat and meat’, ‘Western’, and ‘traditional’ dietary patterns have frequently been associated with a lower education [10,12,14,16], while dietary patterns comprising more healthy food groups, such as ‘vegetable based’, ‘fruit and milk’, ‘plant-based’, or ‘healthy’, are frequently associated with a higher education [9,10,11,14,15,17], although some exceptions have been reported. For example, ‘convenience’ [13], ‘Continental’ [16], or ‘Western’ [47] patterns have been associated with higher education in older adults.

Associations between dietary patterns and alcohol beverage intake are not often examined in older adults, possibly as alcohol beverages are usually included in the dietary pattern as a food group. For example, ‘alcohol and salads’ (REGARDS cohort, USA) [13], ‘Western’ (NutriNet-Sante cohort, France) [10], and ‘Continental’ (Norwegian Breast Screening Programme) [16] patterns had positive loadings (≥ 0.30) for beer, wine, and alcoholic beverages, and negative loadings (≤ −0.30) were reported for wine in a ‘Western’ (Norwegian Breast Screening Programme) [16] pattern. Using a daily alcohol beverage intake, the current study explored associations beyond the alcohol and food correlations found in a dietary pattern to determine whether alcohol was associated with the dietary pattern score in its own right. The ‘prudent’ and ‘Mediterranean’ patterns did not have any significant food group loadings containing alcohol, yet alcohol beverage intake was lower in participants adhering to the ‘prudent’ pattern (*p* < 0.001), and had no association to the ‘Mediterranean’ pattern (*p* = 0.93). Beer loaded significantly on the ‘Western’ pattern (loading = 0.35), and alcohol beverage intake was significantly higher in participants adhering to the ‘Western’ pattern (*p* < 0.001). Two large studies, a multi-ethnic cohort (aged 45–75 years) in the USA [14] and men (aged 40–74 years) in China [15], found higher alcohol intake in participants adhering to ‘fat and meat’, ‘vegetables’, and ‘meat’ dietary patterns, and lower intake in ‘fruit and milk’ and ‘fruit’ patterns.

In the general population, alcohol use and smoking behaviours co-occur regardless of the amount of alcohol consumed [48]. Park et al. [14] and Cai et al. [15] also examined smoking associations and found that, of the five dietary patterns (with an alcohol association), four had parallel associations with smoking. The fifth pattern, ‘vegetables’, had a positive association with alcohol but a negative association with smoking. The current study did not show associations between dietary patterns and smoking (*p* > 0.06), although there was a positive association between alcohol use and smoking (*p* = 0.008, adjusted R^2^ = 0.02).

No associations were observed between dietary patterns and age within the current study. Contrasting results have been reported in other studies in an older population [9,10,12,14,15,16,17,18]. The narrow age band of the REACH study (65 to 74 years) may have precluded observing any associations. The Wellbeing Eating and Exercise for a Long Life (WELL) study, an Australian study in 55 to 65 year olds, reported a ‘red meat, processed meat, white bread and hot chips’ pattern was preferred by the younger men in that cohort [17].

No associations were observed between dietary patterns and the multiple deprivation scores. The Newcastle 85+ study found a ‘low meat’ dietary pattern to be associated with living in an affluent area according to the deprivation index, but this was attenuated when education was included in the model [44]. Our deprivation score is based on residential address, but this has limitations, as several of our participants lived with family, which may not reflect their personal financial status. Another variable used to measure socio-economic factors is income. Other studies in older adults found higher income and education to be associated with healthy food group patterns (‘fruit’ and ‘vegetable’ patterns (males only) [15], and ‘alcohol/salads’ and ‘plant-based’ patterns [13]). In the Three-City and NuAge studies [11], a ‘healthy’ dietary pattern was associated with education but not income.

Our study found that living alone was more prevalent in women than men (χ^2^ = 22.9, *p* < 0.001). Additionally, participants living alone scored low on the ‘Western’ pattern and had no associations with the ‘Mediterranean’ or ‘prudent’ patterns, hence may have a unique dietary pattern not captured by our analysis. Further enquiry may be required to investigate this. Living situations can change in older adults. The death of a spouse can dramatically change a lifestyle from living and sharing meals with someone to learning to cook and shop and eating alone. Living alone does not always mean an absence of nutrition knowledge or desire to eat well [49], as shown in the handful of studies investigating the effect of living alone on dietary patterns in the older adult [10,11,12]. Two ‘healthy’ patterns have been associated with living alone [10,11], and a third study based in the United Kingdom found no association between dietary patterns and living situation [12]. In contrast, living alone has been associated with a higher nutrition risk through a reduced appetite, lower motivation to cook, and preparing simpler meals or perhaps eating more convenient foods [41,50]. This may be more likely for widowers living alone, as their spouse may have shopped and prepared meals [50].

In a New Zealand context, a handful of studies have examined dietary patterns and socio-demographic factors. With regards to education and sex, our findings agree with earlier work by our group [19], where a higher education was positively and negatively associated with ‘healthy’ and ‘traditional’ patterns, respectively, and females were likely to follow the ‘healthy’ pattern, whereas males followed the ‘traditional’ pattern in a representative sample of New Zealand adults (*n* = 4657, aged 15+ years). Alcohol was not considered as a stand-alone variable in that study, but the ‘healthy’ pattern had beer, cider, bitters, wine with a negative load (loading = −0.36), and there were negative associations with smoking and area deprivation [19]. Other New Zealand dietary pattern and socio-demographic studies have primarily been in younger New Zealand women, pre-conception [51] and or in pregnancy [52,53], and in young children [54].

In the current study, socio-demographic and lifestyle factors were associated with 7%, 16%, and 15% of the variation (adjusted R^2^, Table 4) in the ‘Mediterranean’, ‘Western’, and ‘prudent’ patterns, respectively. The education and sex variables and their interaction explained most of the variation in the ‘Western’ pattern, and the negative alcohol beverage intake association explained most of the variation in the ‘prudent’ pattern. Not all studies report the adjusted R^2^ for their multiple regression models. This is unfortunate, as socio-demographic and lifestyle factors do not occur singularly [55,56], and understanding the magnitude of impact for the variety of factors affecting diet and health outcomes can create more efficient and effective public health interventions. For the studies that have reported the variation explained, the R^2^ ranged from 1 to 44% [57,58,59]. Other factors explaining food choice included quality and price of food available, family preferences and taste, trying to eat healthy [60], and physical disability limiting access to food [18,61].

Exploring dietary patterns by nutrient content helps achieve an in depth understanding of the differences between the dietary pattern scores and daily nutrient intake. Additionally, this descriptor may add value if investigating diet–disease associations. As expected, a high fibre intake was associated with a dietary pattern rich in vegetables, fruit, and whole grains, such as the ‘Mediterranean’ and ‘prudent’ patterns. The ‘Mediterranean’ pattern was strongly associated with unsaturated fats and vitamin E, likely from high loadings of nuts, seeds, avocados, olives, and oily fish. The ‘Western’ pattern was strongly associated with energy intake and had small negative associations with fibre, potassium, and magnesium. The ‘prudent’ pattern was associated with a low-fat, high carbohydrate profile, which was supported by high frozen or fresh legumes, and whole grains loadings.

Hu et al. [62] examined the correlations between dietary pattern scores and nutrient intake in their inaugural validation study of dietary patterns. The ‘prudent’ pattern in the Health Professional Follow-up study (males, aged 45–75 years) [62], characterised by vegetables, legumes, whole grains, fruit and fish, had similar nutrient associations to our ‘prudent’ pattern, such as higher fibre and lower total fat. While their ‘Western’ pattern, characterised by processed and red meat, high-fat dairy products, and refined grains, showed similarities to our ‘Western’ pattern, Hu et al. also observed a positive association with total and saturated fats that we did not. The nutrient associations with the 1946 British Birth cohort study (53+ years) [63] patterns ‘health aware’ and ‘refined’ were similar to those for our ‘Mediterranean’ and ‘Western’ patterns. The ‘healthy—France’ and ‘healthy—Quebec’ nutrient patterns (from the Three-City and NuAge studies, aged 65+ years) had similar nutrient contents to the REACH ‘prudent’ pattern, with increased carbohydrate, fibre, iron, and magnesium and reduced saturated and monounsaturated fat intake [64]. Those two patterns also showed increased protein and calcium intake, but these were not apparent for our ‘prudent’ pattern, which showed no or a reduced association with these nutrients.

A major strength of this study is the reproducibility and relative validity of the dietary patterns. The FFQ was validated specifically for dietary patterns [22] as well as nutrient intake [26]. Additionally, a high response rate was achieved, and this study focused on obtaining an in-depth understanding of a specific life stage. Limitations of this study include the subjective decisions required for principal component analysis, such as food grouping, rotation method, number of factors to retain, factor loading interpretation, and naming of dietary patterns. A posteriori dietary patterns are specific to a study population and cannot be generalised. Three dietary patterns (57 food groups) explained 18% of variation in the diet, and minor dietary patterns were not reported, as they were less interpretable and explained a small percent of diet variation. When calculating the nutrient intake, the nutrients allocated to each food item were representative of the food item rather than attempting to capture all foods. Lastly, a convenience sample was used, limiting the variability in some variables e.g., ethnicity and the generalisability of this study’s results. As study participants were volunteers, they may have been more health conscious than the general population, and perhaps this was a reason why two of the three dietary patterns contained healthy foods. Future studies could supplement these findings with additional determinants of diet, such as diet cost and physical functionality, to broaden the dietary pattern picture in the older adult. Additionally, a study in a larger, more representative population group (as demonstrated in Beck et al. [19]) with a wider age range would allow further exploration of ethnicity, living situations, and alcohol consumption.

In conclusion, this paper is the first to investigate dietary patterns in the older New Zealand population. Dietary patterns were associated with socio-demographic and lifestyle factors in the REACH cohort. A ‘Mediterranean’ pattern was associated with being female and having higher physical activity and higher education; a ‘Western’ pattern was associated with being male, having higher alcohol intake, living with others, and having only secondary education; and a ‘prudent’ pattern was associated with higher physical activity and lower alcohol intake. Nutrition policy and public health nutrition should consider associations between dietary patterns and socio-demographic and lifestyle factors. By understanding how these factors influence dietary patterns in the older New Zealand adult, nutrition interventions and health policy can target subsets of the population to perhaps shift people along the scales towards a healthier pattern [19] e.g., men with a only secondary education may need and benefit from specific interventions.

## Figures and Tables

**Table 1:** Food groupings (*n* = 57) used in the dietary pattern analysis.

Food Groups (*n* = 57)	Food Items
Beer	‘Beer, lager, cider (all varieties)’
Other alcohol	‘Port, sherry, liquors’, ‘ready to drink alcoholic beverages’, ‘spirits e.g., gin, brandy, whiskey, vodka’, ‘white wine’
Red wine	‘Red wine’
Bran cereal	‘Bran-based cereals, muesli, porridges—e.g., rolled oats, oat bran, oatmeal, All Bran, Sultana bran’
Refined grains	‘White bread and rolls, including sliced and specialty breads such as foccacia, panini, pita, naan, chapatti, ciabatta, Turkish, English muffin, crumpets, pizza bases, wraps, tortillas, burrito, roti, rewena bread’, ‘white pasta, noodles e.g., spaghetti, canned spaghetti, vermicelli, egg noodles, rice noodles, instant noodles’, ‘white rice’
Snacks	‘Crackers e.g., crisp bread, water crackers, rice cakes, cream crackers, Cruskits, Mealmates, vitawheat’, ‘muesli or cereal bar (all varieties)’
Sweetened cereals	‘Other breakfast cereals e.g., Special K, Light and tasty’, ‘sweetened cereals e.g., Nutrigrain, Fruit Loops, Honey Puffs, Frosties, Milo cereal, CocoPops’, ‘Weetbix, cornflakes or rice bubbles’
Whole grains	‘Brown rice’, ‘couscous, polenta, congee, Bulgur wheat, quinoa e.g., tabbouleh’, ‘whole grain or multi grain bread and rolls including sliced and specialty breads, whole meal or wheat meal bread and rolls including sliced and specialty breads’, ‘whole meal pasta, noodles’
Cheese	‘Cheese e.g., Cheddar, Colby, Edam, Tasty, blue vein, camembert, parmesan, gouda, feta, mozzarella, brie, processed’, ‘cottage cheese, ricotta cheese’
Creamy dairy	‘Cream, sour cream, cream cheese, cheese spreads’
Milk	‘Cow’s milk, including milk as a drink, milk added to drinks (e.g., milky coffees), milk added to cereal’
Other milks (non-dairy)	‘Soy milk, coconut milk, rice milk, almond milk’
Sweetened dairy products	‘Ice cream’, ‘milk-based puddings e.g., rice pudding, custard, semolina, instant puddings, dairy food’, ‘smoothies, milk shakes (made from milk, yoghurt, ice cream), milk shakes, flavoured milk’
Yoghurt	‘Yoghurt’
Dried legumes	‘Beans (canned or dried) e.g., black beans, butter beans, haricot beans, kidney beans, cannellini beans, refried beans, baked beans, chilli beans’, ‘peas and lentils e.g., chickpeas, hummus, falafels, split peas, cow peas, dahl’
Eggs	‘Eggs—boiled, poached, raw’, ‘eggs—fried, scrambled, egg-based dishes including quiche, soufflés, frittatas, omelettes’
Nuts, seeds	‘Nut butters or spreads e.g., peanut butter, almond butter, pesto’, ‘nuts e.g., peanuts, mixed nuts, macadamias, pecan, hazelnuts, brazil nuts, walnuts, cashews, pistachios, almonds’, ‘seeds e.g., pumpkin seeds, sunflower seeds, pinenuts, sesame seeds, tahini’
Soy-based foods	‘Tofu, soybeans, tempeh, vegetarian sausages/meat, vegetarian burger patty, textured vegetable protein’
Oily fish	‘Albacore tuna, salmon, sardines, herring, kahawai, swordfish, carp, dogfish, gemfish, alfonsino, rudderfish, anchovies’, ‘mackerel, snapper, oreo, barracouta, trevally, dory, trout, eel’
Processed fish	‘Crumbed fish e.g., patties, cakes, fingers, nuggets’, ‘fish fried in batter (from fish & chips shop)’
White fish, shellfish	‘Green mussels, squid’, ‘shellfish e.g., cockles, kina, oysters, paua, scallops, shrimp/prawn, pipi, roe’, ‘tuna (canned), hoki, gurnard, hake, kingfish, cod, tarakihi, groper, flounder’
Apples, pears	‘Apples, pears, nashi pears’
Avocados, olives	‘Avocado’, ‘olives’
Bananas	‘Banana’
Berries	‘Strawberries, blackberries, cherries, blueberries, boysenberries, loganberries, cranberries, gooseberries, raspberries (fresh, frozen, canned)’
Citrus fruit	‘Citrus fruits e.g., orange, tangelo, tangerine, mandarin, grapefruit, lemon, lime’
Dried fruit	‘Dried fruit e.g., sultanas, raisins, currants, figs, apricots, prunes, dates’
Other fruit	‘All other fruit e.g., feijoa, persimmon, tamarillo, kiwifruit, grapes, mango, melon, watermelon, pawpaw, papaya, pineapple, rhubarb’
Stone fruit	‘Stone fruit e.g., apricots, nectarines, peaches, plums, lychees’
Poultry	‘Chicken, turkey or duck e.g., roast, steak, fried, steamed, BBQ, casserole, stew, stir fry, curry, mince dishes, frozen dinners’
Processed meat	‘Corn beef (canned), boil up, pork bones, lamb flaps, povi masima’, ‘ham, bacon, luncheon sausage, salami, pastrami, other processed meat’, ‘sausages, frankfurters, cheerios, hot dogs’
Red meat	‘Beef, lamb, hogget, mutton, pork, veal e.g., roast, steak, fried, chops, schnitzel, silverside, casserole, stew, stir fry, curry, BBQ, hamburger meat, mince dishes, frozen dinners’, ‘liver, kidney, other offal (including pate)’
Butter, coconut	‘Butter, ghee’, ‘coconut cream’, ‘coconut oil’
Cakes, biscuits and puddings	‘Biscuits, chocolate or cream filled’, ‘biscuits, plain’, ‘cakes, slices, pastries’, ‘non-milk based puddings e.g., pavlova, sweet pastries, fruit pies, trifle’, ‘pancakes, waffles, sweet buns, scones, sweet muffins, fruit bread, croissants, doughnuts, brioche’
Chocolate	‘Chocolate (all other varieties)’
Confectionery	‘Jam, marmalade, honey, syrups, sweet spreads or preserves’, ‘sugar (all varieties) added to food/drinks’, ‘sweets, lollies’
Salad dressings	‘Creamy dressings e.g., mayonnaise, tartar, thousand island, ranch dressing’, ‘light dressings e.g., French and Italian dressing, balsamic vinegar’
Meat pies, chips	‘Hot potato chips, French fries, wedges’, ‘meat pies, sausage rolls’, ‘potato crisps’
Sauces, condiments	‘Pickles, chutney, mustard’, ‘tomato sauce, barbeque sauce, sweet chilli sauce’, ‘white sauce, cheese sauce, gravies’
Soup	‘Soup, homemade or canned’
Spices	‘Spices e.g., turmeric, ginger, cinnamon’
Vegetable oils	‘Margarine’, ‘vegetable oils’
Yeast spreads	‘Marmite, vegemite’
Diet drinks	‘Diet soft/fizzy drinks e.g., Sprite Zero, Diet Coke, Coke Zero’, ‘low calorie cordials’
Juices	‘Fruit and vegetable juices (all varieties)’
Sugary drinks	‘Cordials including syrups, powders e.g., Raro’, ‘energy drinks e.g., Red Bull, V’, ‘hot chocolate, drinking chocolate, Cocoa, Ovaltine, Nesquik, Milo’, ‘soft/fizzy drinks e.g., Sprite, Coke’, ‘sports drinks e.g., Powerade’
Tea, coffee	‘Coffee (all varieties)’, ‘herbal tea, fruit tea’, ‘tea’
Water	‘Water including tap, bottled or sparkling water’
Alliums	‘Onions, leeks, garlic’
Carrots	‘Carrots’
Cruciferous vegetables	‘Broccoli, cauliflower, Brussel sprouts, cabbage (all varieties)’
Fresh, frozen legumes	‘Green beans, broad beans, runner beans’, ‘peas, green’
Leafy cruciferous vegetables	‘Green leafy vegetables e.g., spinach, silver beet, swiss chard, watercress, puha, whitloof, chicory, kale, chard, collards, chinese kale, bok choy, taro leaves (palusami)’
Other vegetables	‘All other vegetables e.g., corn, pumpkin, mushrooms, capsicum, peppers, courgette, zucchini, gherkins, marrow, squash, asparagus, radish, eggplant, artichoke’
Root vegetables	‘Kumara, taro, green banana, cassava e.g., boiled, mashed, baked, roasted’, ‘other root vegetables e.g., yams, parsnip, swedes, beetroot, turnips’, ‘potato e.g., boiled, mashed, baked, jacket, instant, roasted’
Salad vegetables	‘Salad vegetables e.g., lettuce, cucumber, celery, sprouts’
Tomatoes	‘Tomatoes (all varieties)’

**Table 2:** Participant characteristics.

Characteristic	Total (*n* = 367) Mean ± SD, Median (25, 75) or *n* (%)	Male (*n* = 132) Mean ± SD, Median (25, 75) or *n* (%)	Female (*n* = 235) Mean ± SD, Median (25, 75) or *n* (%)
Age (years) ^‡,^**	69.7 ± 2.6	70.1 ± 2.4	69.4 ± 2.6
Highest level of education ^‡,^***			
Secondary ^a,‡^	83 (23)	18 (14)	65 (28)
Post-secondary	148 (40)	49 (37)	99 (42)
University ^‡^	136 (37)	65 (49)	71 (30)
Employed (paid or volunteer)	179 (49)	55 (42)	124 (53)
Ethnicity			
Asian	11 (3)	5 (4)	6 (3)
Māori/Pacific	10 (3)	5 (4)	5 (2)
NZ European and other	346 (94)	122 (92)	224 (95)
Index of Multiple Deprivation score ^b^	3831 ± 2,766	3943 ± 2,939	3768 ± 2668
Dietary pattern score			
‘Mediterranean’ ^‡,^***	0.00 ± 1.00	−0.22 ± 1.07	0.13 ± 0.94
‘Western’ ^‡,^**	0.00 ± 1.00	0.45 ± 1.10	−0.25 ± 0.84
‘prudent’	0.00 ± 1.00	−0.03 ± 1.20	0.02 ± 0.87
Living situation ^‡,^***			
alone	107 (29)	18 (14)	89 (38)
with others	260 (71)	114 (86)	146 (62)
Physical activity (MET minutes/week) ^c^	3097 (1680, 5118)	3086 (1774, 5464)	3107 (1663, 5037)
Smoker			
Yes (current or past)	78 (21)	29 (22)	49 (21)
No	289 (79)	103 (78)	186 (79)
Daily energy intake (kJ) ^‡,^**	7578 ± 2129	8044 ± 2275	7315 ± 2000
Daily alcohol beverage intake (energy adjusted g/day) ^‡,^***	62 (18, 120)	100 (33, 212)	50 (12, 88)
Food security			
Secure	352 (96)	129 (98)	223 (95)
Moderately secure	13 (4)	2 (2)	11 (5)
Insecure	2 (1)	1 (1)	1 (0)

^‡^ Significant difference between sexes * *p* < 0.05, ** *p* < 0.001, *** *p* < 0.0001; ^a^ No qualification (*n* = 9) and secondary (*n* = 74) aggregated because of small numbers; ^b^ Index of Multiple Deprivation [2], low number = least deprived, range = 11 to 5,636; ^c^ Physical activity MET minutes/week based on 3.3 MET for walking, 4.0 MET for moderate activity, and 8.0 MET for vigorous activity, MET = metabolic equivalence of a task.

**Table 3:** Factor loadings for three major dietary patterns identified using a food frequency questionnaire (*n* = 367).

Food Groups (*n* = 57) ^a,b,c^	Mediterranean	Prudent	Western
Salad vegetables	**0.64**		
Leafy cruciferous vegetables	**0.57**	0.23	
Other vegetables	**0.56**		
Avocados, olives	**0.51**		
Alliums	**0.47**	0.15	
Nuts, seeds	**0.45**	0.26	
White fish, shellfish	**0.45**		
Oily fish	**0.42**		
Berries	**0.41**		
Water	**0.40**	0.18	−0.16
Salad dressings	**0.39**	−0.18	**0.35**
Cruciferous vegetables	**0.39**	0.24	
Eggs	**0.34**		
Cheese	**0.33**	−0.18	**0.34**
Tomatoes	**0.33**		
All other fruit	**0.32**	0.22	
Dried legumes	0.15	**0.68**	
Soy-based foods		**0.65**	
Fresh, frozen legumes		**0.54**	0.20
Whole grains		**0.51**	0.24
Carrots	0.28	**0.48**	
Spices	0.23	**0.30**	
Processed meats		−0.29	**0.59**
Sauces, condiments	0.23		**0.52**
Cakes, biscuits and puddings	−0.26		**0.51**
Meat pies, chips	−0.28		**0.47**
Processed fish			**0.41**
Confectionery	−0.22		**0.39**
Vegetable oils			**0.36**
Beer		−0.21	**0.35**
Chocolate			**0.35**
Sweetened cereal	−0.19		**0.30**
Stone fruit	0.29		0.18
Apples, pears	0.26	0.28	
Dried fruit	0.23	0.25	
Butter, coconut	0.23	−0.20	
Yoghurt	0.19	0.16	
Root vegetables	0.17	0.29	0.24
Red wine	0.15	−0.27	0.16
Refined grains		0.29	0.21
Other milks (non-dairy)		0.28	
Poultry		0.21	0.15
Citrus fruit		0.21	
Bran cereal		0.20	
Bananas		0.17	
Tea, coffee		−0.21	0.21
Other alcohol		−0.21	
Red meat			0.29
Diet drinks			0.28
Sugary drinks			0.25
Milk			0.25
Snacks			0.24
Sweetened dairy products			0.20
Yeast spreads			
Creamy dairy			
Juices			
Soup			
score range	−2.32 to 4.26	−1.93 to 3.83	−2.49 to 8.31
variance explained	7.20	5.30	5.60
Eigenvalue	4.12	3.04	3.18

**^a^ Loadings ≥ 0.30**. A higher loading indicates a greater contribution to the dietary pattern; ^b^ Loadings |< 0.15| excluded for ease in interpretation; ^c^ Positive loadings are positively associated, and negative loadings are negatively associated with the dietary pattern.

**Table 4:** Final models for dietary patterns and socio-demographic and lifestyle factors.

**Mediterranean Pattern**			
**Coefficient**	**Estimate**	**Standard Error**	***p-*** **Value**
Intercept	−0.37	0.14	0.007
Sex male	−0.42	0.11	0.001
Physical activity medium	0.21	0.12	0.097
Physical activity high	0.42	0.12	< 0.001
Education post-secondary	0.39	0.13	0.004
Education university	0.44	0.14	0.002
Reference group (Intercept) is female, low physical activity, and secondary education Adjusted R^2^ = 0.07, *p*-value < 0.001			
**Western Pattern**			
**Coefficient**	**Estimate**	**Standard Error**	***p-*** **Value**
Intercept	−0.37	0.12	0.003
Sex male	1.22	0.25	< 0.001
Education post-secondary	0.13	0.15	0.371
Education university	0.33	0.16	0.035
Living alone	−0.30	0.11	0.006
Alcohol intake	0.00	0.00	0.005
Male: Education post-secondary	−0.86	0.29	0.003
Male: Education university	−0.83	0.29	0.004
Reference group (Intercept) is female, secondary education, living with others, and lower alcohol intake Adjusted R^2^ = 0.16, *p*-value < 0.001			
**Prudent Pattern**			
**Coefficient**	**Estimate**	**Standard Error**	***p-*** **Value**
Intercept	0.13	0.09	0.155
Physical activity medium	0.09	0.12	0.425
Physical activity high	0.37	0.12	0.002
Alcohol intake	−0.00	0.00	< 0.001
Reference group (Intercept) is low physical activity and high alcohol intake Adjusted R^2^ = 0.15, *p*-value < 0.001			
